# Systematic analysis of the frequently amplified 2p15-p16.1 locus reveals PAPOLG as a potential proto-oncogene in follicular and transformed follicular lymphoma

**DOI:** 10.3906/biy-1810-2

**Published:** 2019-04-05

**Authors:** Deniz KURŞUN, Can KÜÇÜK

**Affiliations:** 1 Department of Medical Biology, Faculty of Medicine, Dokuz Eylül University , İzmir , Turkey; 2 İzmir Biomedicine and Genome Center (IBG) , İzmir , Turkey; 3 İzmir International Biomedicine and Genome Institute (iBG-İzmir), Dokuz Eylül University , İzmir , Turkey

**Keywords:** Amplification, 2p15-16, 1, BCL11A, PAPOLG, PUS10, USP34, proto-oncogene, tFL

## Abstract

Transformed follicular lymphoma (tFL) originates from histological transformation of follicular lymphoma (FL), which is the most common indolent non-Hodgkin lymphoma. High-resolution genomic copy-number analysis previously identified frequent amplification of the 2p15-p16.1 locus in FL and tFL cases. The genes (i.e. BCL11A, PAPOLG, PUS10, and USP34) in this amplified locus have not been systematically investigated to date in terms of their role in FL pathogenesis or transformation to tFL. Here we investigated the relationship between amplification and expression of genes in 2p15-p16.1 as well as their expression after histological transformation. NCBI GEO SNP array and gene expression profile (GEP) data of tFL cases were analyzed to evaluate the relationship between amplification and mRNA expression. Moreover, transcript levels of these four genes in FL cases were compared with those of patient-matched tFL cases and normal B-cells. Amplification of the 2p15-p16.1 locus is associated with increased transcription of BCL11A and PAPOLG in tFL cases, of which the latter showed increased expression after histological transformation. Compared with the level in normal B-cells, PAPOLG was significantly overexpressed in FL cases, but expression levels of the other three genes did not show any significant difference. Altogether these results suggest that PAPOLG may be the most critical gene in terms of transformation to tFL.

## 1. Introduction

Follicular lymphoma (FL) is the second most common type of lymphoma in the world (Diumenjo et al., 2016). FL occasionally undergoes histological transformation to higher grade lymphomas such as difuse large B-cell lymphoma (DLBCL) or Burkitt lymphoma (Lossos and Gascoyne, 2011). The characteristic genetic abnormality of FL and transformed follicular lymphoma (tFL) is the t(14;18) chromosomal translocation that leads to constitutive overexpression of BCL2, which promotes survival of germinal center B-cells (Tsujimoto et al., 1985). Recurrent mutations in chromatin regulator genes such as MLL2, EZH2, or CREBBP have been identified to drive FL tumorigenesis (Okosun et al., 2014). Recent studies showed that protection against apoptosis allows earlystage FL cells to proliferate rapidly and acquire additional genetic, epigenetic, and metabolic abnormalities associated with FL pathogenesis (Küppers and Stevenson, 2018; Link, 2018; Minoia et al., 2018). However, the full list of molecular abnormalities related to the development of FL has not been identified or characterized yet.

Histological transformation of FL to higher grade malignancies such as DLBCL has been estimated to occur in ~50% of cases during the course of the disease (Kridel et al., 2012). These tFL cases have a very aggressive clinical course with a median survival time of 1.7 years posttransformation (Al-Tourah et al., 2008) . P53 mutations (Coco et al., 1993) and a pluripotency signature related to MYC overexpression (Gentles et al., 2009) have been identified to be associated with transformation of FL to tFL; however, the full list of molecular aberrations associated with tFL has not been addressed yet.

Genome-wide copy number analysis of FL and tFL cases identified recurrent gain/amplification of the 2p15-p16.1 locus that includes BCL11A, PAPOLG, PUS10, REL, and USP34, which are candidate proto-oncogenes (Eide et al., 2010; Bouska et al., 2014) . A recent report (Hu et al., 2017) showed a weak correlation between REL amplification and its mRNA expression. The same study also showed that ectopic expression of REL marginally promoted cell growth in an FL cell line in limiting serum concentrations. Altogether these observations suggest that other gene(s) located in the amplified 2p15-p16.1 locus may act as proto-oncogene(s) contributing to the development of follicular or transformed follicular lymphoma. To the best of our knowledge, no study has systematically investigated the role of genes other than REL in the 2p15-p16.1 locus on the development of FL or tFL.

Some studies functionally characterized BCL11A as a proto-oncogene in different cancer types. Overexpression of BCL11A has been shown to promote acute leukemia using ex vivo and in vivo experimental models (Yin et al., 2009). In addition, BCL11A has been shown to be an oncogene for triple-negative breast cancer with critical roles in the epithelial stem and progenitor cells (Khaled et al., 2015). Knock-down of BCL11A led to apoptosis of a B-cell lymphoma cell line in the presence of a chemotherapeutic agent (He et al., 2014).

PAPOLG (neo-PAP) was shown to be involved in the posttranscriptional modification of the 3’ ends of transcripts through the addition of adenine nucleotides (Kyriakopoulou et al., 2001), which is critical in terms of mRNA stability and initiation of translation (Yang et al., 2014) . PAPOLG was reported to be overexpressed in some cancer cell types such as breast, colon, ovary, and pancreas (Topalian et al., 2001) , suggesting that it may be a proto-oncogene. It is noteworthy that a mutated version of PAPOLG was described as a tumor-associated antigen that can be recognized by CD4+ T cells in melanoma patients (Topalian et al., 2002).

USP34 plays a critical role in DNA repair in response to double-strand breaks, and it may be involved in the maintenance of genome stability (Sy et al., 2013). USP34 has been shown to promote Wnt/β-catenin signaling, which has an important role in several human cancers (Lui et al., 2011) . PUS10 (DOBI), a novel pseudouridine synthase (McCleverty et al., 2007) , was reported to modify uridine 55 in the TΨC arm of tRNAs (Kamalampeta et al., 2013). Pseudouridine synthases act as RNA chaperones; they facilitate correct folding and assembly of tRNAs (McCleverty et al., 2007).

In the present study, we evaluated the relationship between gene copy number and mRNA expression of BCL11A, PAPOLG, PUS10, and USP34 genes located in the frequently amplified 2p15-p16.1 locus in tFL patient samples, and evaluated whether expression of these genes increases after histological transformation of FL to tFL.

## 2. Materials and methods

### 2.1. Patient samples

The characteristics of the 42 tFL cases used to evaluate the relationship between amplification and mRNA expression of genes located in 2p15-p16.1 were defined previously (Bouska et al., 2014; Hu et al., 2017) . Similarly, the characteristics of the 12 tFL cases whose patientmatched diagnostic pretransformation FL biopsy samples were available were also described previously (Lossos et al., 2002). The ethics committee approval information for the patient samples used in this study was provided in previous studies of the publicly available data. The available ethical committee numbers are as follows: the regional committee of Oslo, Norway, for research ethics (protocol no. S-05 209) and the Institutional Review Board (IRB) of University of Nebraska Medical Center (no: IRB# 513-08-EP). The descriptions for DNA and/or RNA isolation and all subsequent experimental procedures of SNP array or DNA microarray experiments are available in the NCBI Gene Expression Omnibus (GEO) database (accession numbers: GSE67385, GSE81183, GSE3458, and GSE55267). The characteristics of the tFL cases used in this study to evaluate the relationship between gene copy number and expression are shown in Table S1.

### 2.2. Selection of genes in the 2p15-p16.1 locus for copy number and expression analyses

The genes evaluated in the present study, which are located in the frequently amplified 2p15-p16.1 locus, were chosen based on a previous study that comprehensively analyzed copy number alterations in FL and tFL cases (Bouska et al., 2014). The amplified 2p15-p16.1 locus corresponded to recurrent copy number aberration (rCNA) ID: 693, which is a minimal region of abnormality that includes REL, BCL11A, PAPOLG, PUS10, and USP34 genes. As the role of REL in FL/tFL pathobiology was characterized in a recent report (Hu et al., 2017), the other four genes were evaluated in the current study.

### 2.3. Selection of DNA microarray probe sets for gene expression analysis

The sensitivity and specificity values of HG U133 Plus 2.0 DNA microarray probe sets available for the analyzed genes were determined using the GeneAnnot database ( ChalifaCaspi et al., 2003 ). If more than one probe set is available for a gene, then the one with the highest sensitivity and specificity value was chosen for subsequent analyses. If more than one probe set for a gene (e.g., PAPOLG and USP34) has specificity and sensitivity values equal to one, then both probe sets were evaluated in transcript expression analyses.

### 2.4. Gene copy number and transcript expression analysis

**Figure 1:**
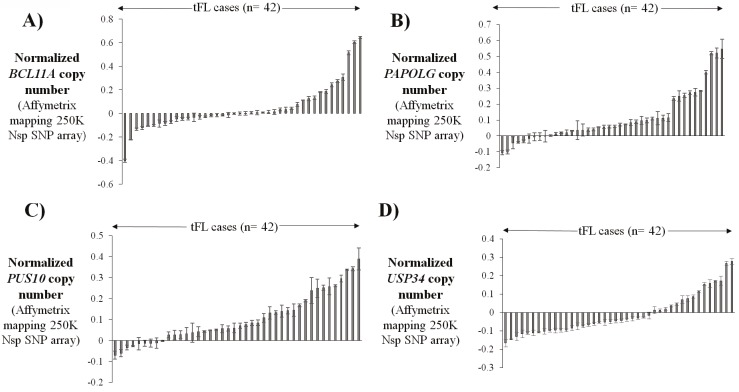
Copy number estimates of four genes located in the 2p15-p16.1 locus in transformed follicular lymphoma cases. Gene copy numbers were estimated by calculating the average SNP array (Affymetrix mapping 250K Nsp SNP array) probe values for BCL11A (A), PAPOLG (B), PUS10 (C), and USP34 (D) for each of 42 tFL cases for which corresponding DNA microarray data are also available. Means ± SD for values of SNP array probes are shown for each gene. SNP probes used to estimate the copy number of each gene are described in the Materials and methods section. SD: Standard deviation.

**Figure 2:**
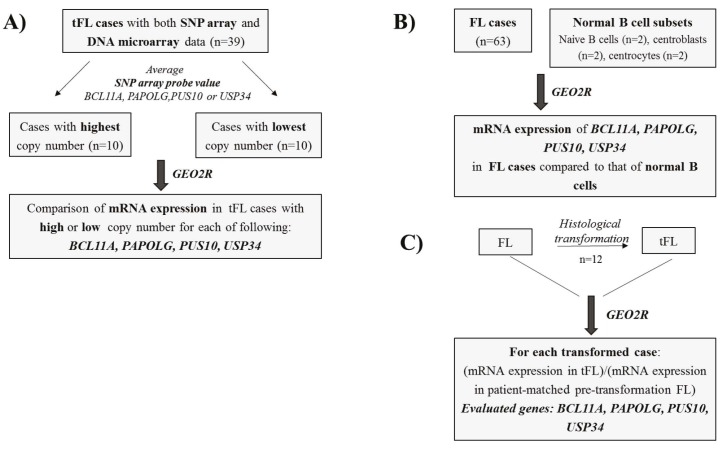
Schematic depiction of the strategies used for mRNA expression analyses of amplified genes in follicular or transformed follicular lymphoma cases. A) Transformed follicular lymphoma cases were divided into two groups based on the high or low copy number of BCL11A, PAPOLG, PUS10, and USP34, which is determined using the values of normalized SNP array data. mRNA expression values were then analyzed with the GEO2R bioinformatics tool using the available DNA microarray probe sets (GEO accession number: GSE81184); B) Transcript expression of four amplified genes was evaluated with the GEO2R tool by comparing the expression levels in FL cases vs. normal B cell subsets (GEO accession number: GSE55267); C) Transcript expression levels of the four amplified genes in 12 tFL cases (i.e. DLBCL) were compared one by one with those of pretransformation levels for each tFL case by using the GEO2R tool (GEO accession number: GSE3458).

Normalized SNP array (Afymetrix Mapping 250 K Nsp SNP Array) probe values of the BCL11A, PAPOLG, PUS10, and USP34 genes were obtained from the NCBI GEO database (accession number: GSE67385). These probe values were determined using the Genotyping Console software (Afymetrix Inc., Santa Clara, CA, USA). Gene copy numbers were estimated by calculating the average value of all corresponding SNP array probes for each gene in 42 tFL samples with available DNA microarray data (Figures 1A-1D). These 42 tFL cases were then divided into two 10-sample groups based on the highest or lowest SNP array probe values for each gene evaluated (i.e. BCL11A, PAPOLG, PUS10, or USP34). After that, the NCBI GEO2R gene expression analysis tool (Barrett et al., 2013) was applied to log2-normalized, median centered Afymetrix Human Genome U133 Plus 2.0 data available as a part of the GSE81183 data set to address whether mRNA expression of each of these four genes differed in tFL cases with low or high gene copy numbers (Figure 2A). The SNP array probe sets evaluated for these genes were as follows: BCL11A: SNP_A-1794265, SNP_A-1813498, SNP_A-2071773, SNP_A-2256538, and SNP_A-4193303; PAPOLG: SNP_A-1892864, SNP_A-2054939, and SNP_A-2094019; PUS10: SNP_A-1808220, SNP_A-2198855, and SNP_A-4208127; USP34: SNP_A-1788538, SNP_A-1845416, SNP_A-1943045, SNP_A- 1956271, and SNP_A-1963530.

### 2.5. Evaluation of transcript levels of the amplified genes in FL cases and normal B cell subsets

The transcript expression level of the genes located in the 2p15-p16.1 amplicon was determined in FL cases (n = 63) and normal B-cell subsets (n = 6) by applying the GEO2R bioinformatics tool to the normalized GEP data set available as a part of the NCBI GEO database (accession number: GSE55267) by comparing the expression levels in FL cases to those in normal B cell subsets (Figure 2B). Similarly, transcript expression levels of these genes in FL cases were compared using GEO2R by dividing the FL cases into low- and high-grade groups (i.e. stage 1–2 vs. stage 3–3a) using the metadata available in GSE55267.

### 2.6. Comparison of mRNA expression levels of genes in the 2p15-p16.1 locus in tFL cases before and after histological transformation

Previously reported gene expression profiles of 12 tFL cases, which were derived from the Lymphochip cDNA microarray platform, were obtained from the NCBI GEO database (accession number: GSE3458). The Lymphochip microarray platform consists of 37,632 hotspots that represent 32,876 unique cDNA clones. In this microarray platform, the posttransformation (i.e. tFL) and the diagnostic FL samples that were obtained from the same patients were labeled with Cy5 or Cy3 uflorescent dye, respectively. For each transcript, the Cy5 to Cy3 ratio was used to calculate the fold-change in expression after histological transformation of FL cases (Figure 2C). Relative transcript expression values from BCL11A cDNA clones (clone IDs: 7940 and 18549), PAPOLG (clone IDs: 845 and 6572), PUS10 (clone IDs: 32285 and 12710), and USP34 (clone IDs: 21935) were determined using the NCBI GEO2R gene expression analysis tool (Barrett et al., 2013) in these tFL cases.

### 2.7. Statistical analysis

A two-sample Student’s t-test was applied to evaluate the statistical significance for the difference observed in mRNA expression of the evaluated genes between tFL cases with amplification and tFL cases without amplification using Microsoft Excel 2016. Any difference with P < 0.05 was considered statistically significant.

## 3. Results

### 3.1. BCL11A and PAPOLG are significantly overexpressed in tFL cases with amplification

**Figure 3:**
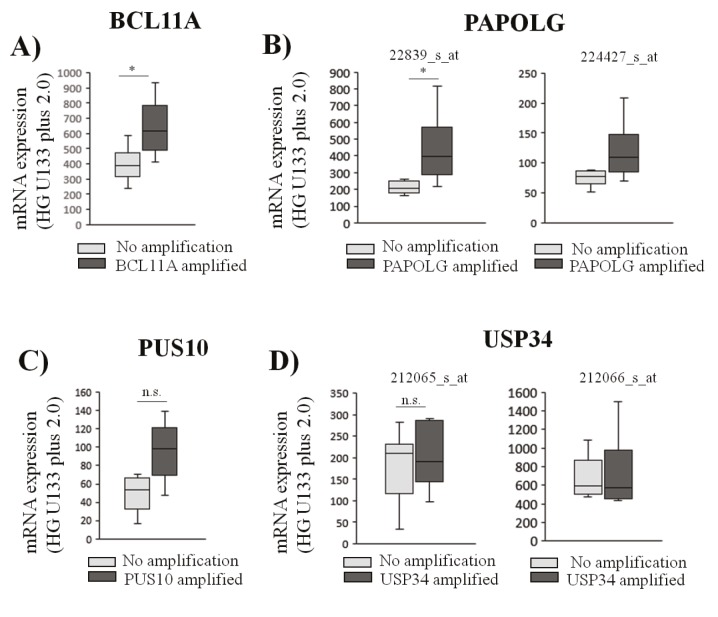
Amplification of BCL11A or PAPOLG is associated with increased transcript expression levels in transformed follicular lymphoma cases. mRNA expression levels of tFL cases with BCL11A (A), PAPOLG (B), PUS10 (C), and USP34 (D) amplifications were compared to those with no amplification of the corresponding gene using probe set values (NCBI GEO dataset: GSE81183) individually and displayed using box-and-whisker plots (t-test, *: P < 0.01). n.s: nonsignificant.

Forty-two tFL cases (Bouska et al., 2014; Hu et al., 2017) were divided into two subgroups based on the average probe intensity values of each of BCL11A, PAPOLG, PUS10, and USP34 to compare expression levels in cases with or without amplification of these genes. Analysis of mRNA expression values using the GEO2R tool showed significantly increased BCL11A mRNA expression in tFL cases with a high copy number compared to cases with a low copy number of the corresponding gene (Figure 3A). Interestingly, PAPOLG showed significantly higher mRNA levels in tFL cases with amplification of PAPOLG (Figure 3B). Transcript expression of PUS10 or USP34 did not show overexpression in the cases with amplification compared to the cases with normal gene copy numbers (Figures 3C and 3D).

### 3.2. PAPOLG and PUS10 mRNA expression increases after histological transformation to tFL

**Figure 4:**
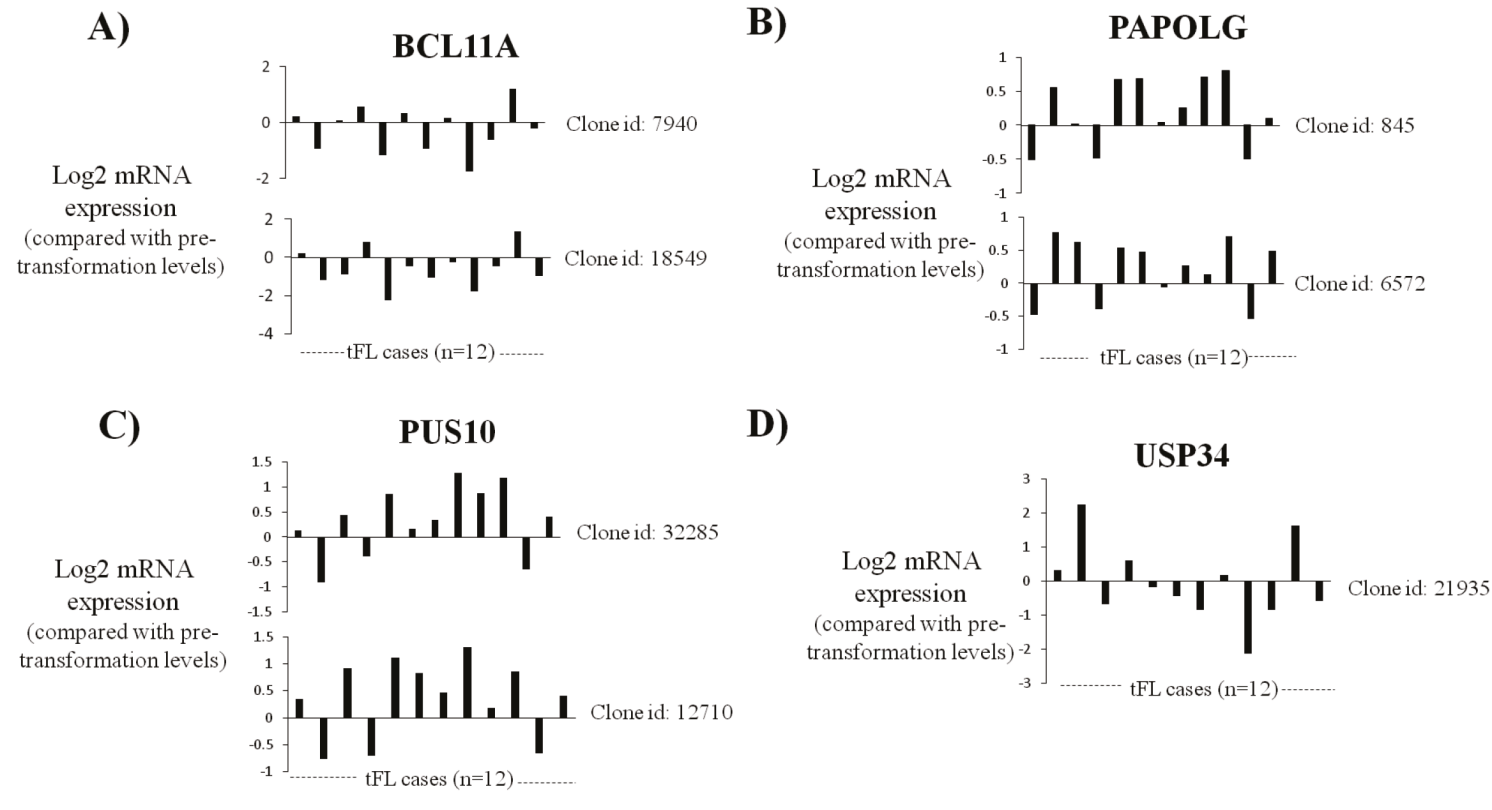
Transcript expression levels of PAPOLG or PUS10 increase in transformed follicular lymphoma tumors compared with those of patient-matched pretransformation tumors. The change in BCL11A (A), PAPOLG (B), PUS10 (C), and USP34 (D) mRNA expression in 12 tFL cases compared to that of pretransformation levels was shown using cDNA microarray clones available in the Lymphochip platform. Clone IDs for each gene are shown near each plot (NCBI GEO accession number: GSE3458).

To address whether expression of genes located in 2p15-p16.1 increases in FL samples after histological transformation to aggressive B-cell lymphoma (e.g., DLBCL), we evaluated whether mRNA expression of genes in 2p15-p16.1 (i.e. BCL11A, PAPOLG, PUS10, and USP34) increased in 12 FL cases after histological transformation to tFL using the NCBI GEO2R bioinformatics tool (Figure 4). We did not observe upregulation of BCL11A mRNA expression in most of the tFL cases analyzed using two different BCL11A cDNA clones (Figure 4A). PAPOLG and PUS10 mRNA expression increased in 75% of FL cases (9 of 12 cases) (Figures 4B and 4C). Most cases did not show upregulation of USP34 expression after histological transformation to tFL (Figure 4D).

### 3.3. PAPOLG is overexpressed in follicular lymphoma cases compared to normal B cells

**Figure 5:**
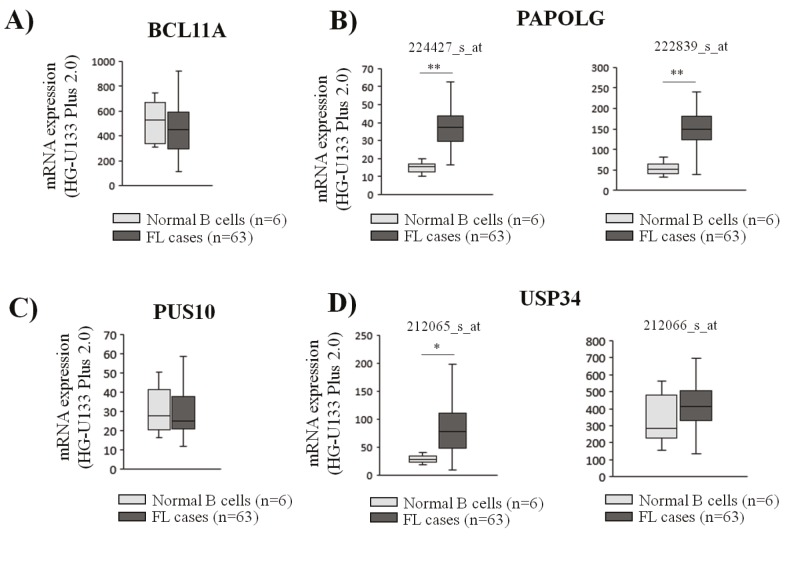
Transcript expression levels of PAPOLG are significantly higher in follicular lymphoma cases compared to the levels in normal B cells. Expression levels of BCL11A (A), PAPOLG (B), PUS10 (C), and USP34 (D) genes were evaluated in FL cases (n = 63) and normal B cell subsets (n = 6) using the GEP data available (GEO accession number: GSE55267). The expression levels of each gene are shown with box-and-whisker plots (t-test, *: P < 0.05, **: P < 0.01).

Next, transcript expression levels of the four amplified genes were evaluated in FL cases (n = 63) and normal B-cell subsets (n = 6) to observe whether their expression is upregulated in FL cases. Of all four evaluated genes, only PAPOLG showed significant upregulation of mRNA expression in FL cases, whereas no significant change was observed for the transcript levels of BCL11A, PUS10, and UPS34 genes (Figures 5A–5D).

### 3.4. Comparison of the expression of amplified genes in low- and high-grade follicular lymphoma cases

**Figure 6:**
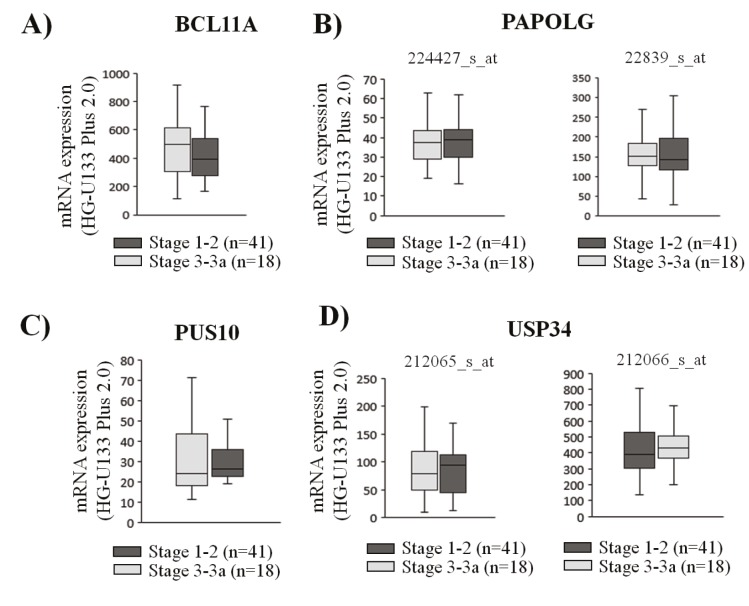
The relationship between expression of amplified genes in low- and high-grade follicular lymphoma cases. Transcript expression levels of BCL11A (A), PAPOLG (B), PUS10 (C), and USP34 (D) in low grade (stage 1–2) and high grade (stage 3–3a) FL cases are shown with box-and-whisker plots.

To address whether any of the amplified genes show higher expression in high-stage FL cases, transcript expression of genes in the 2p15-16.1 locus were determined in 41 high (i.e. stage 3–3a) and 18 low (i.e. stage 1–2) stage FL cases using the GEO2R bioinformatics tool by reanalyzing the GEP data available in GSE55267. No significant difference in gene expression was observed for any of the four evaluated genes in these FL tumor samples (Figures 6A–6D).

## 4. Discussion

Recurrent genomic gains/amplifications may lead to the activation of proto-oncogenes through elevated transcript expression. In most cases, elevated transcript levels lead to increased protein expression of these protooncogenes (Bhargava et al., 2005; Borah et al., 2015) . This amplification-related elevated expression has been shown to promote tumorigenesis in breast cancer cells through constitutive activation of the Akt signaling pathway (She et al., 2008) or chemotherapy resistance in solid tumors such as lung cancer (Engelman et al., 2007) and breast cancer (Li et al., 2010) . Interestingly, amplification of oncogenes such as n-MYC in neuroblastoma (Brodeur et al., 1984) or HER2/NEU amplification in breast carcinoma (Press et al., 1997) was shown to predict poor prognosis in cancer patients. Recurrent gains/amplifications have also been commonly observed in lymphoid malignancies. For instance, amplification of BMI-1, a proto-oncogene involved in regulation of proliferation and senescence (Jacobs et al., 1999), was reported to be associated with high BMI-1 expression in mantle cell lymphoma cases (Bea et al., 2001) . Similarly, another study reported upregulation of the FOXP1 proto-oncogene due to trisomy 3 or focal amplifications in activated B-cell (ABC)-type DLBCL (Lenz et al., 2008) . In particular, recurrent gain/amplification of the 2p15-p16.1 locus, where REL, BCL11A, PAPOLG, PUS10, and USP34 genes reside, is a common observation in different lymphoma types including classical Hodgkin lymphoma (Martin-Subero et al., 2002) and transformed follicular lymphoma (Bouska et al., 2014) .

Our results suggest PAPOLG as a proto-oncogene candidate potentially critical in FL tumorigenesis or histological transformation of FL to tFL due to the following observations. First, gain/amplification of PAPOLG leads to its increased transcript expression in amplified tFL cases. Second, PAPOLG expression increases in most FL cases after transformation to tFL. Third, it is overexpressed in FL cases compared to normal B cell subsets. These observations together with the previous studies that reported overexpression of PAPOLG (neo-PAP) in many cancer types (Topalian et al., 2001) suggest that its overexpression may have critical consequences during the development of FL or tFL through elevated transcript polyadenylation that may result in a more aggressive tumor phenotype as observed for PAP, another poly A polymerase gene, overexpressed in breast cancer (Scorilas et al., 2000) .

In conclusion, systematic analyses of expression of genes located in the recurrently amplified 2p15-p16 locus revealed PAPOLG as the most likely candidate protooncogene in development of FL and transformation of FL to tFL. These analyses provide the basis for functional assays in the future that will be needed to elucidate the role of PAPOLG in these malignancies.

## Acknowledgment

We thank the Young Scientists Award Program of the Turkish Academy of Sciences (TÜBA GEBİP 2017) (C.K.) for supporting this study.
